# Supplementary material for: A New Bioassay Platform Design for the Discovery of Small Molecules with Anticancer Immunotherapeutic Activity
Source: Mar Drugs. 2020 Nov 29;18(12):604. doi: 10.3390/md18120604 (PMC7760914; doi:10.3390/md18120604)
Supplement: Supplementary file 1 [file marinedrugs-18-00604-s001.pdf]

Supplemental Information

A new bioassay platform design for the discovery of lead compounds with anticancer and immunomodulant activity

Figure S1

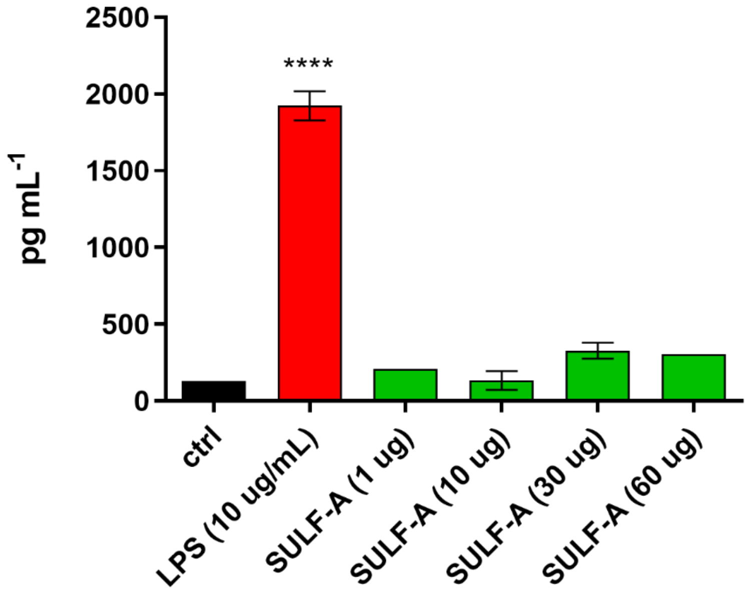

Figure S1. TNF-α production (pg mL^{-1}) measured on D1 cell supernatants in untreated cells (ctrl) and cells treated with LPS and Sulfavant A (SULF-A) in the range of concentration from 1 to 60 µg mL^{-1}. The assay was performed by TNF-α ELISA kit (Thermo Scientific).

Figure S2

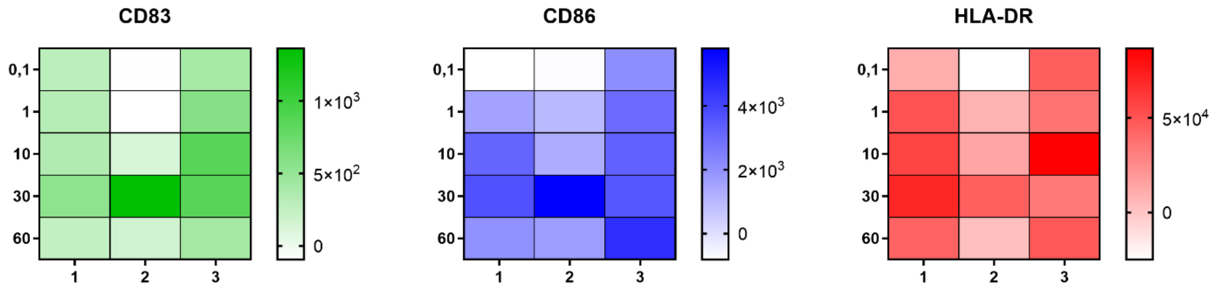

Figure S2. Surface expression analysis of CD83, CD86 and HLA-DR in moDCs from 3 donors (1,2,3) treated with Sulfavant A at the concentrations showed on the y axis (µg/mL); All data were normalized to the cells treated only with the vehicle (MeOH). The color bar on the right shows the MFI (Mean Fluorescence Intensity) measured for each marker.

Figure S3

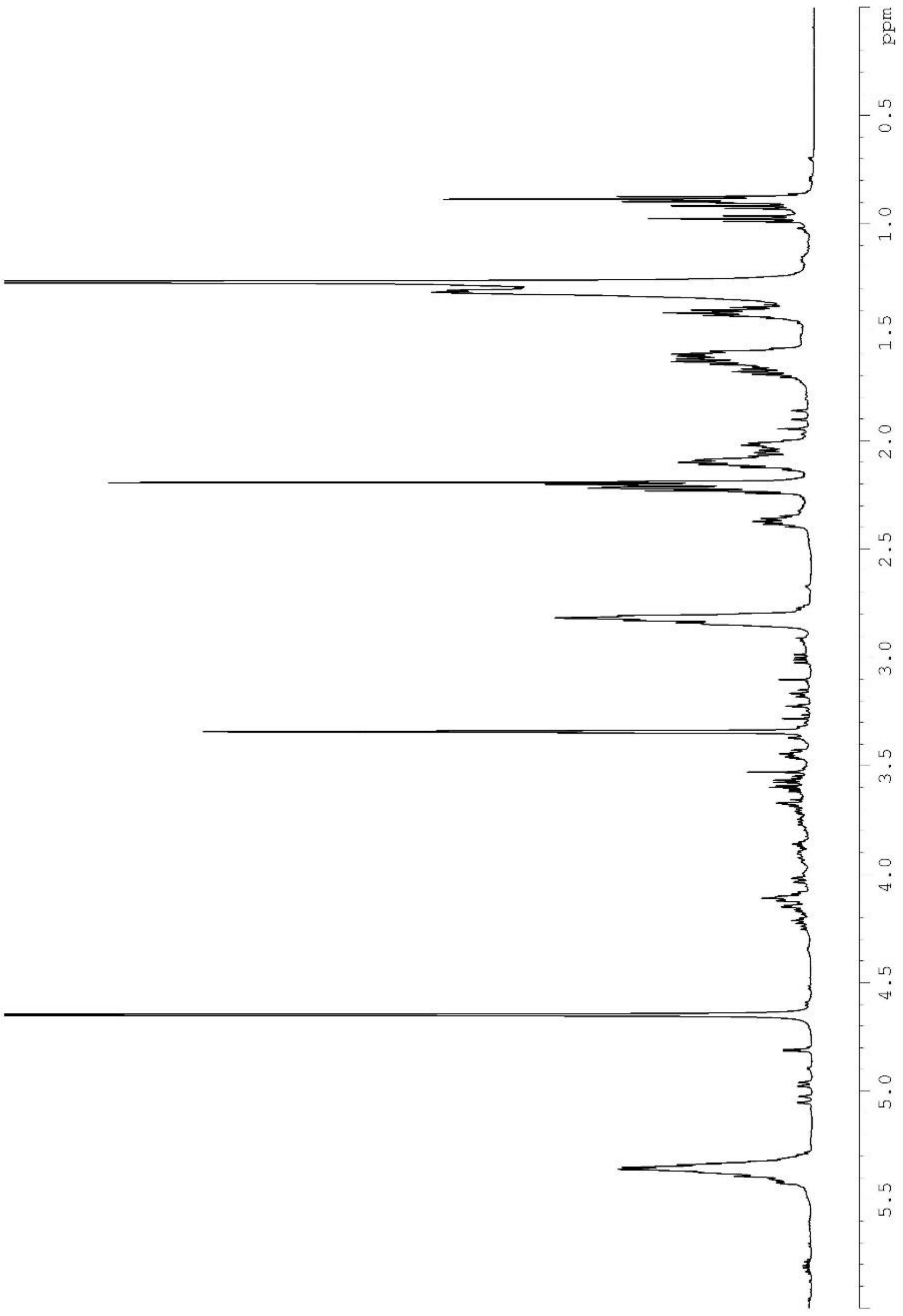

Figure S3. 1H-NMR spectrum (600MHz, CD3OD:$CDCl_3$ 1:1 v: v) of active C fraction obtained by HR-X fractionation of *Thalassiosira weissflogii* extract, that showed an enrichment of α-SQDG (Manzo et al., 2017).

Figure S4

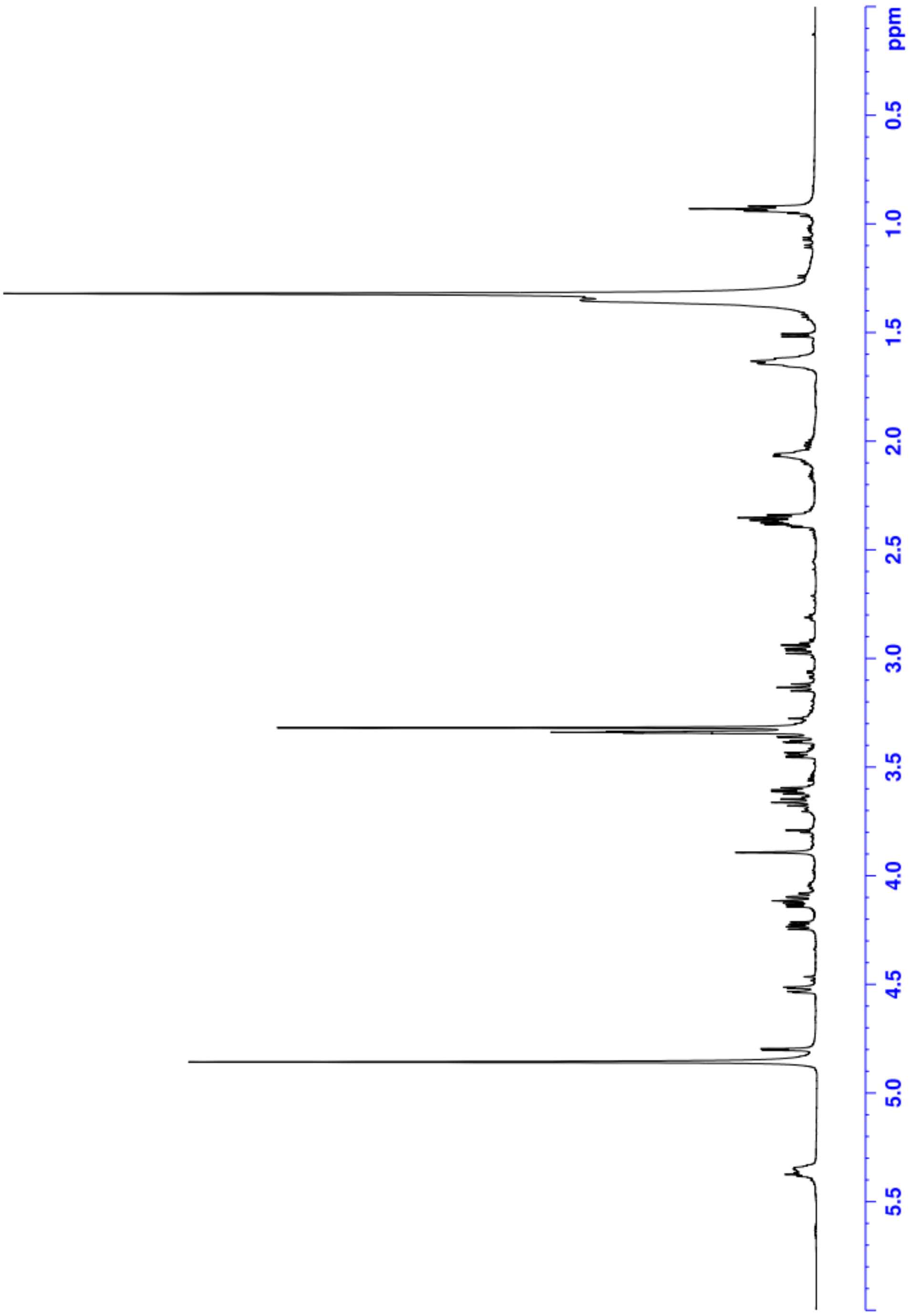

Figure S4. 1H-NMR spectrum (600MHz, CD_3OD) of α-SQDG from Thalassiosira weissflogii.
